# Aiming for a negative fluid balance in patients with acute lung injury and increased intra-abdominal pressure: a pilot study looking at the effects of PAL-treatment

**DOI:** 10.1186/2110-5820-2-S1-S15

**Published:** 2012-07-05

**Authors:** Colin Cordemans, Inneke De laet, Niels Van Regenmortel, Karen Schoonheydt, Hilde Dits, Greg Martin, Wolfgang Huber, Manu LNG Malbrain

**Affiliations:** 1 Department of Intensive Care, Ziekenhuis Netwerk Antwerpen, ZNA Stuivenberg, Campus Stuivenberg, Lange Beeldekensstraat 267, 2060, Antwerpen 6, Belgium; 2 Grady Memorial Hospital, Emory University School of Medicine, Atlanta, GA, USA; 3 II. Medizinische Klinik, Klinikum Rechts der Isar, Technische Universität München, Munich, Germany

**Keywords:** abdominal pressure, extravascular lung water, fluid balance, fluid management, capillary leak, organ failure, treatment, conservative late fluid management, albumin, PEEP.

## Abstract

**Introduction:**

Achievement of a negative fluid balance in patients with capillary leak is associated with improved outcome. We investigated the effects of a multi-modal restrictive fluid strategy aiming for negative fluid balance in patients with acute lung injury (ALI).

**Methods:**

In this retrospective matched case-control study, we included 114 mechanically ventilated (MV) patients with ALI. We compared outcomes between a group of 57 patients receiving PAL-treatment (PAL group) and a matched control group, abstracted from a historical cohort. PAL-treatment combines high levels of positive end-expiratory pressure, small volume resuscitation with hyperoncotic albumin, and fluid removal with furosemide (Lasix^®^) or ultrafiltration. Effects on extravascular lung water index (EVLWI), intra-abdominal pressure (IAP), organ function, and vasopressor therapy were recorded during 1 week. The primary outcome parameter was 28-day mortality.

**Results:**

At baseline, no significant intergroup differences were found, except for lower PaO_2_/FIO_2 _and increased IAP in the PAL group (174.5 ± 84.5 vs 256.5 ± 152.7, *p *= 0.001; 10.0 ± 4.2 vs 8.0 ± 3.7 mmHg, *p *= 0.013, respectively). After 1 week, PAL-treated patients had a greater reduction of EVLWI, IAP, and cumulative fluid balance (-4.2 ± 5.6 vs -1.1 ± 3.7 mL/kg, *p *= 0.006; -0.4 ± 3.6 vs 1.8 ± 3.8 mmHg, *p *= 0.007; -1,451 ± 7,761 vs 8,027 ± 5,254 mL, *p *< 0.001). Repercussions on cardiovascular and renal function were limited. PAL-treated patients required fewer days of intensive care unit admission and days on MV (23.6 ± 15 vs 37.1 ± 19.9 days, *p *= 0.006; 14.6 ± 10.7 vs 25.5 ± 20.2 days, respectively) and had a lower 28-day mortality (28.1% vs 49.1%, *p *= 0.034).

**Conclusion:**

PAL-treatment in patients with ALI is associated with a negative fluid balance, a reduction of EVLWI and IAP, and improved clinical outcomes without compromising organ function.

## Introduction

Both early and late fluid management affect outcome in acute lung injury (ALI), sepsis, and trauma [1-5]. After initial filling to reverse distributive shock [6], emphasis shifts to limitation and elimination of interstitial edema in vital organs. Indeed, a positive fluid balance resulting from third spacing is independently associated with impaired organ function and worse outcome [7-11]. Conversely, achievement of negative fluid balances predicts survival and improves lung function [12,13].

Bedside measurement of extravascular lung water index (EVLWI) performed by transpulmonary thermodilution allows to estimate the extent of capillary leak and fluid overload [14-17]. Accordingly, EVLWI correlates well with organ function and survival [15,16,18,19]. Moreover, fluid management aimed at EVLWI reduction results in a more negative fluid balance and improved outcomes [20]. In order to achieve a negative fluid balance, previous prospective trials excluded patients with hypotension and renal failure [12,20,21].

In this study, we aimed for a negative fluid balance in mechanically ventilated patients with ALI presenting with severe hypoxemia, increased EVLWI, and intra-abdominal pressure (IAP) using a restrictive fluid management, referred to in our institution as "PAL-treatment". PAL-treatment combines high levels of positive end-expiratory pressure (PEEP), small volume resuscitation with hyperoncotic albumin, and fluid removal with furosemide (Lasix^®^) or ultrafiltration during continuous renal replacement therapy (CRRT).

## Methods

### Study design

In this retrospective matched case-control single center study, patients with PAL-treatment were compared to a matched control group for 1 week from the onset of ALI. Outcomes were assessed at day 28 after enrolment or at the day of death or hospital discharge, whichever occurred first. The primary outcome parameter was hospital mortality. Secondary outcomes included intensive care unit (ICU) and hospital length of stay, development of intra-abdominal hypertension (IAH), duration of mechanical ventilation (MV) and cumulative fluid balance, organ dysfunction, and vasopressor therapy requirements after 1 week.

### Patients

Data of 114 patients treated in two ICU's in Ziekenhuis Netwerk Antwerpen, ZNA Campus Stuivenberg, Antwerp, Belgium were collected from March 2004 to August 2007 (control group) and from March 2008 to February 2010 (PAL group). Patients were consecutively included if they were intubated and MV and if monitoring with transpulmonary thermodilution technique was performed.

The PAL group consisted of 57 patients with ALI according to international criteria [22], in whom a negative daily fluid balance was deemed necessary, according to clinical appraisal of low P_a_O_2_/FIO_2 _ratio and increased EVLWI and IAP. All patients were included at the onset of ALI.

The historical cohort consisted of 123 MV patients with thermodilution catheter monitoring, of which 65 patients met the criteria of ALI [22] and had data available from the onset of ALI. We used an automatic case-control matching software module to abstract 57 control patients from this group. In this way, controls were matched to PAL-treated patients with regard to demographics, etiology of lung injury, severity of illness, organ dysfunction, fluid balance, and EVLWI at baseline.

### Treatment protocol

Approval for this epidemiologic study was granted by our institutional review board (EC approval number 3766). Due to the observational and retrospective character of this study, informed consent was waived. Standard treatment was based on recent ICU guidelines and did not differ between the two groups.

Patients in the PAL group received a combination therapy aiming for negative daily fluid balances. First, application of PEEP was titrated to counterbalance increased IAP (best PEEP in cmH_2_O = IAP in mmHg). Next, hyperoncotic albumin (20%) solution was administered by 200-ml boluses over 60 min twice on the first day and subsequently titrated toward a serum albumin level of 30 g/L. Finally, a furosemide drip was initiated with an intravenous loading dose of 60 mg, followed by a continuous infusion at 60 mg/h for the first 4 h and 5-10 mg/h thereafter, according to hemodynamic tolerance. In anuric patients, CRRT was initiated with an ultrafiltration rate resulting in neutral to negative daily fluid balances.

### Data collection

Demographic, clinical, and laboratory data were registered in an electronic database, supplemented with daily fluid balance, sepsis-related organ failure assessment (SOFA) score, IAP, MV settings, and hemodynamic variables. Finally, data on total duration of MV, CRRT, ICU stay, hospital stay, and mortality on day 28 were added to the database.

Capillary leak index (CLI) was defined as C-reactive protein (CRP; milligrams per deciliter) over albumin (grams per liter) ratio, multiplied by 100 [23]. Requirement of vasopressor therapy was determined by the need of norepinephrine with a dose ≥ 0.1 μg/kg/min.

Severity of illness on ICU admission was described by an averaged simplified acute physiology (SAPS II) score [24], acute physiology and chronic health evaluation (APACHE II) score [25], and SOFA score [26]. Daily fluid balance was calculated by subtracting the fluid output (diuresis, ultrafiltration volume in case of CRRT, and any loss from drainage tubes) from the fluid intake (IV and enteral fluid administration); each day the cumulative fluid balance was computed by the addition of daily fluid balances.

IAP was the mean of two daily IAP measurements via a Foley bladder catheter, as described previously [27]. IAH was defined as persistent increase of IAP ≥ 12 mmHg and abdominal perfusion pressure (APP) as mean arterial pressure (MAP) minus IAP according to consensus definitions [8].

A central venous catheter and a thermistor-tipped arterial thermodilution catheter (Pulsiocath 5F) inserted into the femoral artery and attached to a PiCCOplus^® ^system (Pulsion Medical Systems, Munich, Germany) were already in place for each patient. Transpulmonary thermodilution measurements were obtained by central venous injection of three 20-mL boluses of cooled saline (< 8°C). For each set of thermodilution determinations, the mean values were used for statistical analysis. Cardiac output (CO), global end diastolic volume (GEDV), extravascular lung water (EVLW), global ejection fraction (GEF), pulmonary vascular permeability index, stroke volume variation (SVV), and pulse pressure variation were calculated using the PiCCOplus^® ^[17]. EVLW was indexed to body weight (EVLWI) and CO and GEDV to body surface area (cardiac index (CI), GEDVI).

### Statistical analysis

A priori analyses were performed to stratify patient groups by demographics, etiology of lung injury, severity of illness, organ dysfunction, fluid balance, and EVLWI at baseline. We analyzed data on intent-to-treat basis comparing outcomes on different time points within groups and between groups during 1 week.

Continuous data were expressed by mean ± standard deviation (SD), and intergroup differences were determined by one-way analysis of variance (ANOVA) analyses day by day for 1 week (univariate analysis). Categorical data were expressed as frequency distributions and/or percentages, and the *χ*^2 ^test was used to determine intergroup differences. Two-sided *p *values of 0.05 or less were considered to indicate statistical significance.

Time course of PEEP, albumin, P_a_O_2_/FIO_2 _ratio, EVLWI, daily and cumulative fluid balance, SOFA score, and IAP was described by clustered error bar graphs representing mean ± standard error. The Kaplan-Meier method was used to analyze differences in cumulative survival and duration of mechanical ventilation. We used SPSS software package (version 17.0.1; SPSS, Chicago, IL, USA). Automatic case-control matching was performed with the fuzzy extension (http://www.spss.com/devcentral).

## Results

### Baseline characteristics

We included 114 mainly medical (*n *= 102) mechanically ventilated patients with ALI. Fifty-five patients (48.2%) required vasopressor therapy, and 50 patients (43.9%) received CRRT at baseline.

The PAL group had on average lower PaO_2_/FIO_2 _(higher respiratory SOFA score), increased IAP, and higher PEEP level. Otherwise, the two groups were similar (Table 1).

**Table 1:** Baseline characteristics

Variable	Control group (*n *= 57)	PAL group (*n *= 57)	*p *value
Age (year)	61.4 ± 16.8	63.0 ± 14.3	0.598

Male sex (%)	73.7	66.7	0.539

BMI	25.2 ± 4.0	26.1 ± 6.0	0.366

Primary lung injury (%)			0.607
Sepsis	43.9	47.4	
Pneumonia	22.8	26.3	
Aspiration	12.3	8.8	
Burns	5.3	7.0	
Trauma	7.0	3.5	
Other	8.8	7.0	

Medical ICU (%)	87.7	91.2	0.344

Severity of disease			
SAPS II	52.3 ± 17.3	47.9 ± 18.4	0.188
APACHE II	22.7 ± 11.1	22.9 ± 11.4	0.934
SOFA score			
Respiratory	1.9 ± 1.4	2.4 ± 1.3	0.037
Coagulation	0.9 ± 1.2	1.0 ± 1.2	0.488
Liver	0.6 ± 1.0	0.8 ± 1.2	0.302
Cardiovascular	2.9 ± 1.5	3.0 ± 1.2	0.640
Nervous	2.5 ± 1.7	2.6 ± 1.6	0.867
Renal	1.5 ± 1.6	1.5 ± 1.6	0.864
Total	10.2 ± 4.2	11.3 ± 4.0	0.160
Number of organs failing	2.1 ± 1.1	2.5 ± 1.2	0.061
Hemodynamic variables			
HR (bpm)	98.0 ± 18.0	96.5 ± 19.4	0.733
Mean arterial pressure (mmHg)	83.3 ± 13.5	84.9 ± 11.0	0.502
Vasopressor use (%)	58.0	52.0	0.688
CI (L/min/m^2^)	3.4 ± 0.9	3.8 ± 0.9	0.145
SVV (%)	15.5 ± 8.4	12.0 ± 5.5	0.088
GEF (%)	19.7 ± 6.6	22.1 ± 7.6	0.260
GEDVI (mL/m^2^)	736.8 ± 141.5	807.5 ± 189.3	0.163
EVLWI (mL/kg)	12.0 ± 6.1	13.4 ± 6.2	0.326
Respiratory variables			
Tidal volume (mL/kg of PBW)	8.6 ± 1.8	7.9 ± 1.9	0.119
Plateau pressure (cmH_2_O)	25.1 ± 9.1	25.3 ± 7.4	0.914
PEEP (cmH_2_O)	6.7 ± 2.4	10.2 ± 2.9	< 0.001
Dynamic compliance (mL/cmH_2_O)	39.4 ± 17.4	42.8 ± 22.4	0.402
PaO_2_/FIO_2_	256.5 ± 152.7	174.5 ± 84.5	0.001
Renal and metabolic variables			
Creatinine (mg/dL)	2.1 ± 2.2	1.8 ± 1.5	0.393
Urine output (mL/day)	1,366 ± 1,273	1,591 ± 1,139	0.323
CRRT (%)	47.4	40.4	0.571
Albumin (g/L)	25.3 ± 8.0	26.7 ± 6.6	0.297
pH	7.33 ± 0.12	7.35 ± 0.11	0.430
Immune system			
CRP (mg/dL)	14.7 ± 13.5	15.3 ± 10.1	0.806
Central nervous system			
Glasgow Coma Score	8.2 ± 5.4	7.6 ± 5.1	0.557

Capillary leak index	68.9 ± 66.3	62.9 ± 48.4	0.589

Intra-abdominal pressure (mmHg)	8.0 ± 3.7	10.0 ± 4.2	0.013

Abdominal perfusion pressure (mmHg)	75.3 ± 14.0	75.1 ± 12.9	0.933

Fluid balance day before enrolment (mL)	2,504 ± 2,704	1,659 ± 4,419	0.224

BMI, body mass index; ICU, intensive care unit; SAPS, simplified acute physiology score; APACHE, acute physiology and chronic health evaluation; SOFA, sepsis and organ failure assessment; HR, heart rate; CI, cardiac index; SVV, stroke volume variation; GEF, global ejection fraction; GEDVI, global end diastolic volume index; EVLWI, extravascular lung water index; PEEP, positive end-expiratory pressure; CRRT, continuous renal replacement therapy; CRP, C-reactive protein; PBW, predicted body weight; HR, heart rate.

### Direct treatment effects (Figure 1 and Table 2)

Serum albumin concentration averaged 26.0 ± 7.4 g/L in the total group. From day 2, patients in the PAL group had significantly higher concentrations, rising to 33.0 ± 7.3 g/L on day 7 (*p *< 0.001). Controls had no significant increase in albumin after 1 week (25.3 ± 8.0 vs 26.4 ± 5.0 g/L, *p *= 0.399).

**Figure 1:**
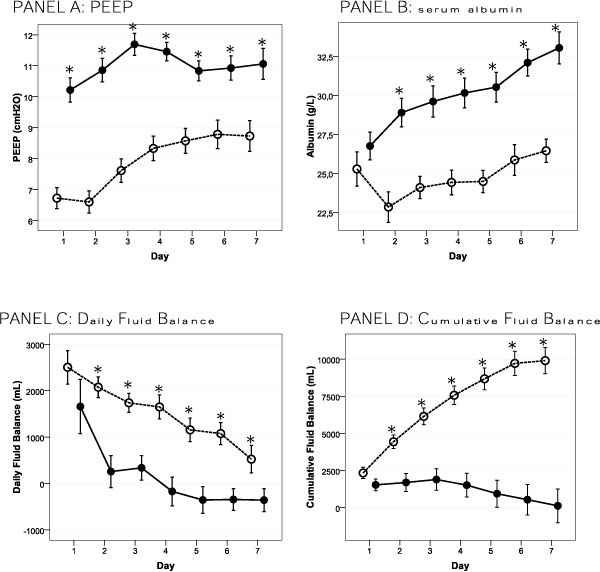
**Mean ± standard error of variables directly affected by 1 week of PAL-treatment**. PAL-treated patients are depicted by a full line and control patients by a dotted line. *p < 0.05, day-by-day pairwise compared between the PAL group and the control group (one-way ANOVA).

**Table 2:** Mean absolute change of selected variables after 1 week

Variable	Control group	PAL group	*p *value
SOFA score			
Respiratory	0.0 ± 1.7	-0.8 ± 1.6	0.015
Coagulation	0.4 ± 1.1	0.1 ± 0.9	0.181
Liver	0.3 ± 1.1	0.4 ± 0.8	0.553
Cardiovascular	-0.5 ± 1.9	-1.2 ± 2.0	0.087
Nervous	0.2 ± 2.0	-0.2 ± 1.8	0.271
Renal	0.2 ± 1.8	0.3 ± 1.5	0.693
Total	0.7 ± 5.4	-1.3 ± 5.0	0.057

Number of organs failing	-0.1 ± 1.6	-0.6 ± 1.5	0.115

Hemodynamic variables			
HR (bpm)	-11.5 ± 16.3	11.1 ± 95.2	0.183
Mean arterial pressure (mmHg)	4.1 ± 13.6	0.6 ± 15.5	0.247
CI (L/min/m^2^)	0.9 ± 0.2	1.0 ± 0.2	0.274

Respiratory variables			
Plateau pressure (cmH_2_O)	2.2 ± 8.6	0.7 ± 8.9	0.431
PEEP (cmH_2_O)	2.1 ± 3.6	0.5 ± 3.8	0.050
Dynamic compliance (mL/cmH_2_O)	1.6 ± 24.3	5.3 ± 31.4	0.548
PaO_2_/FIO_2 _ratio	-12.3 ± 166.4	99.9 ± 110.5	< 0.001

EVLWI (mL/kg)	-1.1 ± 3.7	-4.2 ± 5.6	0.006

Albumin (g/L)	1.1 ± 9.0	6.3 ± 8.9	0.008

Creatinine (mg/dL)	-0.5 ± 2.0	-0.1 ± 1.1	0.171

Capillary leak index	-17.1 ± 75.5	-31.0 ± 47.4	0.111

Intra-abdominal pressure (mmHg)	1.8 ± 3.8	-0.4 ± 3.6	0.007

Abdominal perfusion pressure (mmHg)	0.4 ± 14.4	1.3 ± 15.5	0.785

Cumulative fluid balance (mL/day)	8,027 ± 5,254	-1,451 ± 7,761	< 0.001

SOFA, sepsis and organ failure assessment; HR, heart rate; CI, cardiac index; PEEP, positive end-expiratory pressure; EVLWI, extravascular lung water.

CLI was significantly reduced during 1 week of PAL-treatment (62.9 ± 48.4 vs 31.9 ± 25.5, *p *< 0.001), in contrast to non-significant reductions in the control group (68.9 ± 66.3 vs 51.8 ± 42.5, *p *= 0.139).

In the PAL group, average PEEP for the entire week was significantly higher (11.0 ± 2.8 vs 7.9 ± 2.9 cmH_2_O, *p *< 0.001) compared to controls. PEEP level correlated with average IAP of 10.0 ± 3.4 mmHg (*R *= 0.293, *p *< 0.001).

After the day of enrolment, patients in the PAL group had significantly lower daily fluid balances on each day; a negative daily fluid balance was achieved on average by day 4. PAL-treated patients had a trend toward higher average urine output (1,844 ± 1,714 vs 1,681 ± 1,635 mL, *p *= 0.182). Resulting cumulative fluid balance after 1 week was significantly higher in the control group.

### Effects on organ function (Figure 2 and Table 2)

Respiratory function improved significantly in the PAL group. The PaO_2_/FIO_2 _ratio increased from 174.5 ± 84.5 to 274.4 ± 116.9 (*p *< 0.001) after 1 week in PAL-treated patients but remained unchanged in the control group (256.6 ± 152.7 vs 244.2 ± 98.3, *p *= 0.641). Accordingly, respiratory SOFA score after 1 week decreased only in the PAL group. EVLWI was reduced from 12.0 ± 6.1 to 10.9 ± 3.2 mL/kg (*p *= 0.021) in the control group and from 13.4 ± 6.2 to 9.2 ± 3.6 mL/kg (*p *= 0.006) in the PAL group. Except for a higher setting of PEEP, respiratory mechanics were similar between groups at all time points.

**Figure 2:**
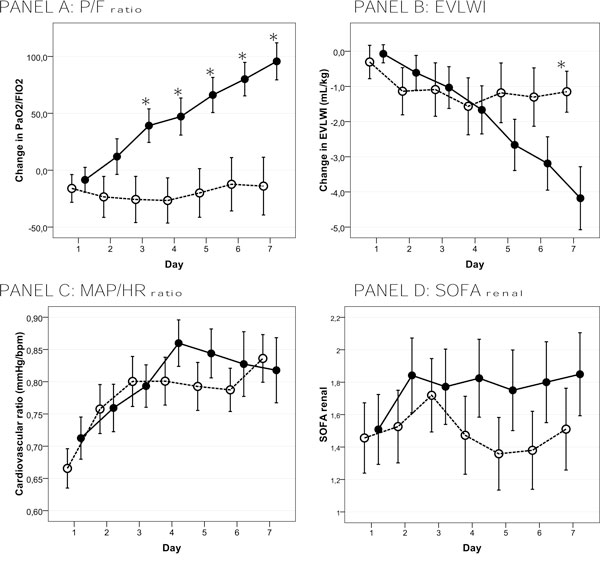
**Assessment of respiratory, cardiovascular, and renal functions**. Mean ± standard error for change to baseline in respiratory function (reflected by P/F ratio and EVLWI), cardiovascular function reflected by MAP/heart rate ratio, and renal function as assessed with renal SOFA score during 1 week of PAL-treatment. PAL-treated patients are depicted by a full line and control patients by a dotted line. *p < 0.05, day-by-day pairwise compared between the PAL group and the control group (one-way ANOVA).

Overall hemodynamic impact assessed with cardiovascular SOFA score shows a significant improvement after 1 week in the PAL group (3.0 ± 1.2 vs 1.8 ± 1.6, *p *< 0.001) and a modest but insignificant decrease in the control group (2.9 ± 1.5 vs 2.4 ± 1.5, *p *= 0.099). Time course of the MAP/heart rate ratio is shown in Figure 2. Other hemodynamic monitoring variables were not significantly affected.

After 1 week, a higher percentage of patients required vasopressor therapy in the PAL group (Table 3). Furthermore, average dose of norepinephrine for the entire week was greater in the PAL group (0.281 ± 0.284 vs 0.180 ± 0.129 μg/kg/min, *p *= 0.005).

**Table 3:** Major outcomes

	Control group	PAL group	*p *value
Death at day 28 (%)	49.1	28.1	0.034
ICU stay (day)	37.1 ± 19.9	23.6 ± 15	0.006
Hospital stay (day)	82.5 ± 57.6	69.8 ± 66.9	0.475
Vasopressor therapy after 1 week (%)	30.6	60.8	0.003
Duration mechanical ventilation (day)	25.5 ± 20.2	14.6 ± 10.7	0.020
Duration CRRT (day)	6.2 ± 8.8	10.0 ± 3.8	0.437

ICU, intensive care unit; CRRT, continuous renal replacement therapy.

Changes in renal function as assessed with renal SOFA score were similar in both groups. However, average serum creatinine during the observed week was higher in PAL-treated patients (1.9 ± 3.3 vs 1.6 ± 1.3 mg/dL, *p *= 0.038) (Table 2).

**Figure 3:**
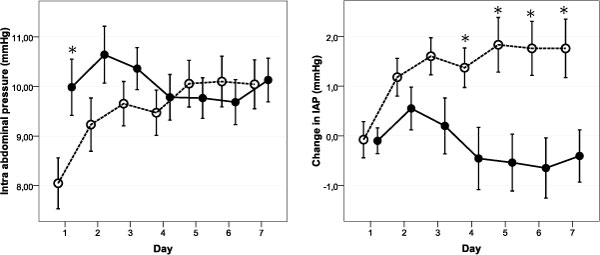
**Mean ± standard error for intra-abdominal pressure during 1 week of PAL-treatment**. IAP increases significantly in control patients and remains stable in PAL-treated patients. According to PAL-treatment protocol, PEEP level was titrated to IAP (best PEEP equals IAP) resulting in a mean PEEP of 11.0 ± 2.8 cmH_2_O. PAL-treated patients are depicted by a full line and control patients by a dotted line. *p < 0.05, day-by-day pairwise compared between the PAL group and the control group (one-way ANOVA).

As shown in Table 2 and Figure 3, IAP increased in the control group but remained stable in the PAL group. The APP remained unchanged during the whole week. During observation, 33.6% of patients developed IAH, 39.6% in the PAL group, and 27.8% in the control group (*p *= 0.224).

### Major outcomes (Table 3 and Figure 4)

A total of 44 patients (38.6%) died, with more deaths in the control group than in the PAL group (49.1 vs 28.1%, *p *= 0.034). Patients in the PAL group surviving their ICU stay required fewer days of ICU admission and days on MV. Total hospital stay and days with CRRT were similar in both groups.

**Figure 4:**
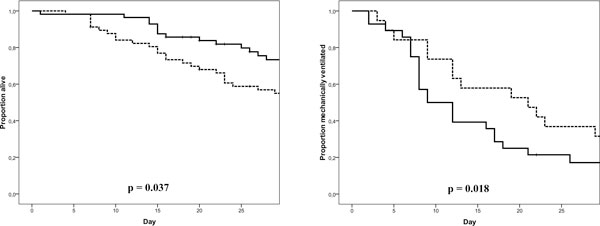
**Kaplan-Meier plot for cumulative survival and days on mechanical ventilation**. PAL-treated patients are depicted by a full line and control patients by a dotted line.

## Discussion

In this study, we demonstrated that a multi-modal approach using PAL-treatment in patients with ALI achieves negative cumulative fluid balance without compromising organ function. Furthermore, compared to a matched control group, we found improved oxygenation, EVLWI and IAP reduction, fewer days on mechanical ventilation, shorter ICU stay, and reduced 28-day mortality in the PAL group.

The idea behind PAL-treatment aiming for negative fluid balance in a setting of capillary leak is based on the recently rediscovered concept of the ebb and flow phase [2,13,28,29]. The ebb phase represents a distributive shock characterized by increased capillary permeability and albumin leak [2,28,30]. Excess interstitial fluid leads to organ dysfunction [31], including ALI, secondary IAH [8], and associated acute kidney injury [32]. Shock reversal and subsequent hemodynamic and renal recovery sets in the transition to the flow phase resulting in mobilization of excess extravascular (lung) water [2]. Previously, a neutral to negative cumulative balance [1,7,10,13,33,34] and reduction of EVLWI were shown to correlate with improved survival [15,16,18,19].

PAL-treatment intends to initiate the flow phase, limiting capillary leak and promoting interstitial fluid removal while ensuring organ perfusion at the same time. Therefore, it is a specific form of restrictive fluid management, combining open lung ventilation, small volume resuscitation with hyperoncotic albumin, and aggressive fluid removal.

Open lung ventilation strategy in ALI signifies application of high levels of PEEP [35] and is correlated with decreases in EVLWI [36]. Both the percentage of potentially recruitable lung and EVLWI are related to outcome [15,16,18,19,35]. The open lung strategy in ALI is associated with increased alveolar fluid clearance and reduced EVLWI [37-39]. In this study, PAL-treatment was initiated in patients with low oxygenation index and high EVLWI, potentially indicating a higher proportion of recruitable lung. Within the concept of the polycompartment syndrome, we set PEEP level (cmH_2_O) equal to IAP (mmHg) in order to counteract IAP [40] (Figure 3).

Induction of the flow phase with PAL-treatment implies vascular refilling from the interstitium and subsequent removal of fluids from the body producing a net negative fluid balance. In this context, addition of small volume resuscitation with hyperoncotic albumin to a fluid removal regimen resulted in a greater negative fluid balance while maintaining better hemodynamic stability [21]. Moreover, restoration of colloid osmotic pressure in absence of elevated hydrostatic pressure may prevent further interstitial edema formation [41,42]. Accordingly, hypoproteinemia is highly predictive of positive fluid balance and development of ALI in patients with sepsis [43]. In view of PAL-treatment as a therapy for capillary leak, beneficial effects of albumin on the microcirculation may be of particular interest, attenuating capillary permeability and pulmonary inflammation [44-47].

PAL-treatment proved to be effective to achieve negative fluid balance. After 1 week, PAL-treated patients had a net negative cumulative fluid balance while control patients added up to a positive cumulative fluid balance, similar to other cohorts [12,20]. In contrast to previous studies, negative fluid balance was pursued as a specific goal. One week of PAL-treatment did not significantly worsen cardiovascular function. Yet, more patients required vasopressor therapy and administered doses were higher. Furthermore, although renal SOFA score on each day was similar in both groups, the PAL group had higher average creatinine for the observed week. In line with previous reports, successful restrictive fluid strategy with PAL-treatment led to improved oxygenation and shortened duration of MV [12,21]. Moreover, PAL-treated patients had a significant greater reduction of EVLWI. This observation possibly reflects improved healing of lung injury, better shock reversal with transition to the flow phase [15,16,18,19]. We found PAL-treated patients to have a significant reduction of CLI as a result of restoration of serum albumin.

Overzealous fluid therapy in a setting of capillary leak is an important risk factor for IAH, associated with organ failure and increased mortality [48]. Therefore, a fluid strategy aimed at negative fluid balance and avoiding crystalloid over-resuscitation may play an important role in preventing and even treating IAH [49]. In this context, our observations demonstrated a significant increase of IAP in controls, whereas IAP dropped in PAL-treated patients.

Our study has several important limitations. First, the use of historical controls may raise difficulties to ensure that obtained differences in outcome are related to the studied treatment. Indeed, we cannot deny that continuous evolving standard care has led to better outcomes in patients with acute lung injury [50]. In particular, there are indices that a more protective ventilation (not only higher PEEP as per protocol) was applied in the PAL group since at baseline controls had slightly higher tidal volumes and lower PEEP levels. However, as patients were selected for PAL-treatment based on low oxygenation indices and high EVLWI, they may have had a higher percentage of potentially recruitable lung, requiring higher PEEP levels [35].

Second, the large difference in mortality between the two groups has to be placed in context. Expected mortality in the control group and PAL group was 47% and 48%, computing a standardized mortality ratio of 1.04 and 1.70, respectively. Apart from the presumed better standard care over time, a selection bias may have been introduced by including only mechanically ventilated patients with thermodilution catheter monitoring. Thus, we selected a specific case mix of severely ill ALI patients prone to exhibit fluid retention, in which attention to fluid balance may be expected to have great potential benefit. In this regard, we note a considerable high cumulative fluid balance after 1 week in controls (8,027 mL), albeit similar to earlier reports [12,20]. Third, since this was an open trial in which fluid therapy decisions were made by the treating physician, the lack of a strict protocol to guide fluid therapy may have introduced bias. Fourth, our database did not supply detailed information on amounts of fluids administrated in the first 6 h. The fluid balance on the day before enrolment was almost 1 L higher in controls, possibly indicating a more aggressive initial volume replacement. Exact data on the type of fluid used and the rate of hourly ultrafiltration in patients with CRRT were not recorded either.

## Conclusion

PAL-treatment in patients with ALI results in a negative cumulative fluid balance, a reduction of EVLWI and IAP, and improved clinical outcomes. Repercussions on cardiovascular and renal function were limited. Within the concept of dual response to inflammatory injury, we conclude that PAL-treatment could safely and effectively promote the transition to flow phase. Future double-blinded trials confirming these observations and investigating PAL-treatment in other settings of capillary leak are warranted.

## Abbreviations

ALI: acute lung injury; ANOVA: analysis of variance; APACHE: acute physiology and chronic health evaluation; APP: abdominal perfusion pressure; CI: cardiac index; CLI: capillary leak index; CO: cardiac output; CRP: C-reactive protein; CRRT: continuous renal replacement therapy; EVLW(I): extravascular lung water (index); GEDV(I): global end diastolic volume (index); GEF: global ejection fraction; IAH: intra-abdominal hypertension; IAP: intra-abdominal pressure; ICU: intensive care unit; MAP: mean arterial pressure; MV: mechanical ventilation; PAL-treatment: PEEP + albumin + Lasix (furosemide); PEEP: positive end-expiratory pressure; SAPS: simplified acute physiology score; SOFA: sepsis and organ failure assessment; SVV: stroke volume variation; vs: versus.

## Competing interests

GM, WH, and MM are members of the medical advisory board of Pulsion Medical Systems (Munich, Germany), a monitoring company. The other authors declare that they have no competing interests.

## Authors' contributions

CC, IDL, NVR, KS, HD, and MM planned the study and were responsible for the design, coordination, and drafting the manuscript. GM and WH participated in the study design and helped to draft the manuscript. CC and MM performed the statistical analysis and helped to draft the manuscript. All authors read and approved the final manuscript.
